# Improved feasibility of astronaut short-radius artificial gravity through a 50-day incremental, personalized, vestibular acclimation protocol

**DOI:** 10.1038/s41526-020-00112-w

**Published:** 2020-08-26

**Authors:** Kathrine N. Bretl, Torin K. Clark

**Affiliations:** University of Colorado Boulder, 3775 Discovery Drive, Boulder, CO 80309 USA

**Keywords:** Aerospace engineering, Biological sciences

## Abstract

The “Coriolis” cross-coupled (CC) illusion has historically limited the tolerability of utilizing fast-spin rate, short-radius centrifugation for in-flight artificial gravity. Previous research confirms that humans acclimate to the CC illusion over 10 daily sessions, though the efficacy of additional training is unknown. We investigated human acclimation to the CC illusion over up to 50 daily sessions of personalized, incremental training. During each 25-min session, subjects spun in yaw and performed roll head tilts approximately every 30 s, reporting the presence or absence of the illusion while rating motion sickness every 5 min. Illusion intensity was modulated by altering spin rate based upon subject response, such that the administered stimulus remained near each individual’s instantaneous illusion threshold. Every subject (*n* = 11) continued to acclimate linearly to the CC illusion during the investigation. Subjects acclimated at an average rate of 1.17 RPM per session (95% CI: 0.63–1.71 RPM per session), with the average tolerable spin rate increasing from 1.4 to 26.2 RPM, corresponding to a reduction in required centrifuge radius from 456.6 to 1.3 m (to produce loading of 1 g at the feet). Subjects reported no more than slight motion sickness throughout their training (mean: 0.92/20, 95% CI: 0.35–1.49/20). We applied survival analysis to determine the probability of individuals reaching various spin rates over a number of training days, providing a tolerability trade parameter for centrifuge design. Results indicate that acclimation to a given, operationally relevant spin rate may be feasible for all subjects if given a sufficient training duration.

## Introduction

For decades, artificial gravity (AG) has been proposed as the most comprehensive spaceflight countermeasure, holding the potential to concurrently protect multiple physiological systems from in-flight, microgravity-induced deconditioning. Several conceptual designs have been proposed, although all face the challenge of budgetary and engineering constraints. The most feasible approach to AG in the near term is thought to be short-radius, intermittent centrifugation^1^, though additional challenges surface with this design. Of these, it appears the human body can acclimate quite quickly to Coriolis forces^2^ and gravity gradients^3,4^, but acclimation to the disorienting “Coriolis” cross-coupled (CC) illusion is less expeditious and thorough (we note that the CC illusion is not the result of a conventional and substantial Coriolis effect and therefore have placed it in quotations to distinguish from traditional Coriolis forces). It remains unclear whether all individuals can tolerate and/or acclimate to the moderately high spin rates (e.g., ~15+ rotations per minute (RPM)) that would be required for short-radius centrifugation.

The CC illusion is a provocative tilting or tumbling sensation experienced by a subject in a constantly rotating field after he/she performs a head tilt out of the plane of constant rotation. The illusion is highly disorienting and typically leads to motion sickness^5^. The intensity of the illusion is proportional to the spin rate at which the subject is exposed, the magnitude of the head tilt, and the velocity of the tilt^6,7^. Higher spin rates are required for effective short-radius centrifuge designs; thus, the CC illusion becomes increasingly relevant as the potential for disorientation and motion sickness becomes more prominent with a faster-spinning centrifuge.

Previous research has shown that humans are capable of acclimating to the CC illusion, as subjects reported reduced intensity of the illusion after two or three sessions of exposure^8–11^. These early acclimation studies exposed subjects to the CC illusion at spin rates of 23 RPM or higher. This very provocative stimulus resulted in substantial motion sickness in subjects; notably, it caused ~25–35% of subjects to dropout of each study due to motion sickness, despite the fact that subjects who were highly susceptible to motion sickness were often excluded from participating^8,10,11^.

Subsequent studies investigated the possibility of benign exposure to the CC illusion. It was found that an incremental protocol provides a less provocative way to expose subjects by reducing motion sickness via incremental spin rate exposure (i.e., increasing spin rates over time rather than directly exposing subjects to a high CC illusion stimulus/fast spin rate). One incremental CC illusion acclimation approach exposed subjects to spin rates of 14 RPM on day 1, 23 RPM on day 2, and 30 RPM on day 3^12^. This study showed that the incremental steps improved subjects’ tolerance of the acclimation protocol, yet it was still too aggressive for some of the subjects, as 14% (4/28) of the subjects dropped out due to motion sickness. Another variant of incremental CC illusion acclimation showed that personalized, incremental acclimation over 2 days of training resulted in essentially no cases of motion sickness, and therefore, a 0% subject dropout rate (0/8 subjects)^13^.

We have recently extended previous efforts to investigate personalized, incremental acclimation to the CC illusion over ten consecutive weekdays^14^. In short, subjects were seated upright and spun in yaw about an Earth-vertical axis. After an introduction to the CC stimulus, each subject began the training protocol spinning at 1 RPM. While spinning at a constant rate, subjects performed roll head tilts (40° right ear down and back to upright) approximately every 30 s. After each head tilt, subjects reported if they experienced the CC illusion. If the subject did not report the illusion on two consecutive head tilts of one head tilt pair (head tilt down and back upright), the spin rate was increased by 1 RPM; otherwise, the spin rate was maintained. Ultimately, the spin rate was incremented only based upon subject response, and the stimulus was always just barely noticeable (i.e., personalized and threshold based). The stimulus was effective in acclimating all 10 subjects, while not being excessively strong to elicit substantial motion sickness. Subjects acclimated from an average threshold (i.e., fastest spin rate at which no CC illusion was felt) of 1.8 RPM (range: 1–3 RPM) before any training to 17.7 RPM (range: 3–30 RPM) after 10, 25-min training sessions. Across all sessions and subjects, an average motion sickness rating of only 1.06/20 was reported. This investigation confirmed that a personalized, incremental protocol is a highly effective, benign method of acclimating individuals to the CC illusion.

In addition to personalized acclimation, we have also investigated the efficacy of standardized (i.e., incremental, but non-personalized) acclimation^15^ and the ability of subjects to retain gained acclimation after a period without training^16^. These studies have suggested that non-personalized acclimation is not as effective or tolerable as personalized acclimation; however, acclimation in both studies appears to be mostly retained for up to 90 days of unmonitored activity. These previous studies sought to investigate acclimation after an initial protocol of 10 daily sessions. Remarkably, it appeared acclimation continued linearly over those 10 days, suggesting further acclimation may be possible. However, potential for acclimation beyond these 10 days is unknown. Specifically, it is of operational and scientific interest to determine if acclimation continues with additional exposure, or if a plateau is reached in some or all individuals, after which no further acclimation is possible.

The objective of this investigation is to test the ability of subjects to acclimate to extended CC illusion exposure (i.e., up to 50, 25-min sessions), using essentially the same personalized, incremental staircase described above, and inform operational considerations of centrifuge design.

## Results

### Extended acclimation protocol resulted in continued acclimation with no evidence of plateau in all subjects

**Fig. 1:**
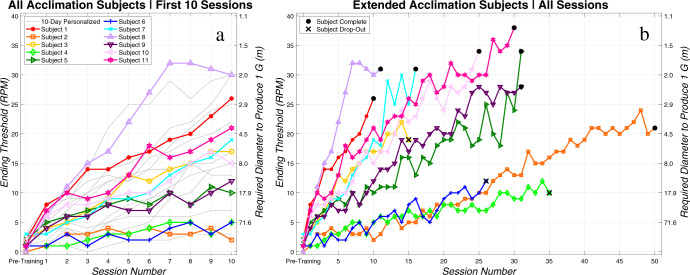
Extended acclimation findings. CC illusion acclimation pretraining and ending thresholds for (**a**) 10-day and extended acclimation subjects over the first 10 sessions and (**b**) extended acclimation subjects over the entire duration of their involvement in the study. In panel **a**, the previous 10-day investigation subjects are shown in gray, while the current extended acclimation subjects are overlaid in color. In panel **b**, subjects who completed the study by reaching the investigation’s ending criteria are shown with a black circle at their final data point, while subjects who left the study due to scheduling challenges are denoted with a black “X”.

Eight of our 11 subjects completed the investigation by reaching one of our first two ending criteria (see “Methods”): either a beginning threshold (i.e., fastest spin rate at which no illusion was experienced at the beginning of the session) of 25 RPM (n = 7) or completing a total of 50 testing sessions (*n* = 1). Notably, none of the subjects appeared to have reached a plateau in ending threshold based on our ending criteria (Fig. 1). The final three subjects who enrolled in the study were included in analysis, as they each completed 15 or more testing sessions. However, they did not complete the study protocol; all three chose to prematurely leave the study due to challenges with scheduling (denoted with a black “X” in Fig. 1b). Including all 11 subjects, the average number of training sessions completed was 25.5 (range: 10–50 sessions). Subjects began the investigation at an average pre-training threshold (i.e., the fastest spin rate at which no illusion was felt before any acclimation training) of 1.4 RPM (range: 0–3 RPM, a threshold of 0 RPM corresponds to reporting the illusion at 1 RPM). While acclimating over a varying number of sessions, subjects reached an average ending threshold (i.e., fastest spin rate that elicited no CC illusion at the conclusion of the session) of 25.8 RPM (range: 10–38 RPM) on their final day of testing. This resulted in a significant increase in CC illusion acclimation threshold (paired *t*-test: *t*(10) = 8.95, diff = 24.4 RPM, Cohen’s d = 3.73, *p* < 0.0005). Subjects’ acclimation can also be quantified by their acclimation rate (i.e., the slope of a linear fit to each subject’s ending threshold as a function of session number). Average acclimation rate was found to be significantly non-zero across subjects (One-Sample *t*-test: *t*(10) = 4.82, mean = 1.17 RPM per session, 95% CI: 0.63–1.71 RPM per session, *p* = 0.0007). Further, all 11 subjects exhibited positive acclimation rates (range: 0.26 RPM per session to 2.91 RPM per session), showing that all subjects—even those who increased their threshold at a slower pace—were able to acclimate to the CC illusion.

### Extended acclimation investigation subjects with modified staircase protocol and previous 10-day subjects acclimated similarly over first 10 days

These findings are similar to what has been previously reported over just 10 days^14^, although additional confidence has been gained with the extended exposure. Figure 1a shows our original 10-day personalized acclimation subjects in gray with our extended acclimation subjects overlaid in color (for just the first 10 days of the investigation). This plot shows that the extended acclimation subjects performed within the same bounds as our previous 10-day acclimation subjects, despite the slight modifications in the staircase protocol with the inclusion of catch trials and the option to increment to slower spin rates if the CC illusion was reported on each head tilt for three consecutive head tilt pairs (see “Methods”). A two-sample *t*-test investigating potential differences between ending thresholds on day 10 of the two subject groups confirmed the two groups were comparable, with a non-significant result (Two-sample independent *t*-test: *p* = 0.46). Additionally, there was no evidence to suggest a difference in acclimation rate between the extended acclimation subjects and the previous 10-day acclimation subjects over the first 10 days of the investigation (Two-sample independent *t*-test: *p* = 0.27).

All subjects acclimated to the CC illusion, but as displayed in Fig. 1, they acclimated at substantially different rates. As observed in previous studies^13–16^, there is a large degree of inter-individual differences in acclimation. As one measure in the present study, the acclimation rate averaged 1.17 RPM per session but varied substantially with a standard deviation of 0.81 RPM per session, yielding a coefficient of variation of 0.69.

### Subjects reported low motion sickness throughout study

**Fig. 2:**
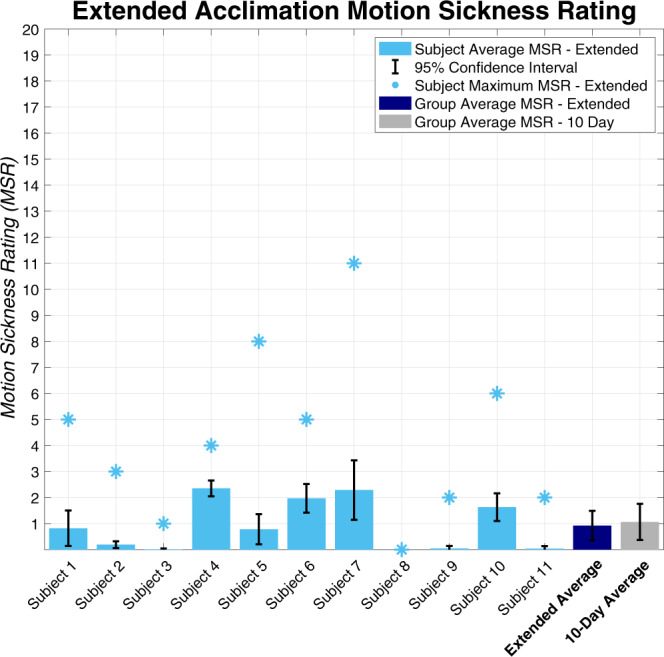
Extended acclimation motion sickness results. Subject averages are denoted with the light blue bars, maximum reports across all sessions are shown with light blue asterisks, and the dark blue bar shows the overall average across all extended duration acclimation subjects. The gray bar shows the 10-day personalized subject group average motion sickness score for comparison. Error bars show 95% confidence intervals about the subject means.

The incremental, personalized protocol was designed to limit motion sickness experienced during acclimation. The protocol was effective in providing this benign acclimation to the CC illusion, as subjects reported generally low motion sickness scores throughout the investigation (Fig. 2).

Most importantly, none of the 11 subjects dropped out of the study due to excessive motion sickness, nor was any session terminated early due to subject discomfort or elevated motion sickness reporting (i.e., 10/20 or higher on the utilized 0–20 scale, where 0 corresponds to no motion sickness and 20 corresponds to extreme nausea or vomiting). We note that one subject did report an 11/20 on one of the sessions, although this report was communicated in the final moments of the session, therefore the complete session was conducted. The average MSR across all subjects and sessions was 0.92 (95% CI: 0.35–1.49) on the commonly used 0–20 scale. This was not significantly different than the average MSR of 1.06/20 (95% CI: 0.37–1.76) from the 10 subjects who completed the previous 10-day personalized protocol (Mann–Whitney–Wilcoxon test, *p* = 0.78).

We observed no trend in MSR reporting across sessions or reports within a session; however, we did find a statistically significant correlation between subjects’ average reported MSR and their MSSQ percentile (Spearman Rank Correlation: *t*(9) = 3.35, R = 0.75, *p* = 0.008). This suggests that subjects who are more susceptible to motion sickness (based on our screening before the experiment began) experienced and reported higher motion sickness levels during the investigation.

### High reliability of subject reporting of subjective CC illusion

In order to assess reliability of subject reporting, we randomly included up to one “catch trial” per session, which was either well above or well below the subject’s instantaneous CC illusion threshold (see “Methods”). A total of 245 catch trials were completed across all subjects and sessions; 132 (53.9%) of the trials were High Catch Trials (50% faster spin rate), and 113 (46.1%) were Low Catch Trials (50% slower spin rate, 35 trials which would have been Low Catch Trials were not performed due to the rules outlined in “Methods”). Overall, subjects almost always responded to the catch trials as we would have expected. Specifically, on only 4.5% of the catch trials did the subject respond unexpectedly: either that they felt the illusion on both head tilts for a Low Catch Trial (2.4%) or that they did not feel the illusion during a High Catch Trial (2.0%). This gives us confidence that the subjects were reporting reliably and were able to maintain consistent criteria of what qualifies as the presence of the CC illusion throughout the duration of the study.

### Survival analysis and expected population acclimation

With this study and those published previously, we have shown that subjects who undergo the developed training protocol become more tolerant of the CC illusion over time, though subjects acclimate at varying rates. To use this research as a tool for centrifuge design, we have conducted an analysis to estimate the probability that individuals will acclimate to a specific spin rate over a number of training days. Since we found no statistical differences between the data collected during the previous 10-day acclimation study (*n* = 10) and the current extended acclimation study (*n* = 11), we pooled these data to improve the precision of the analysis.

**Fig. 3:**
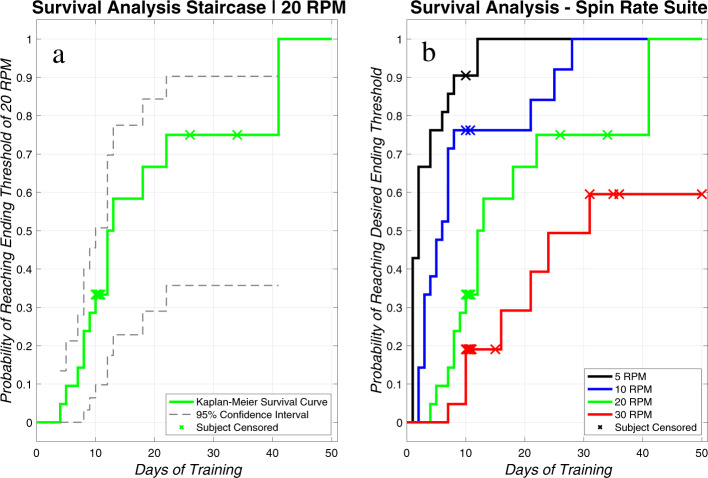
Staircase of survival analysis findings. Survival analysis results for (**a**) 20 RPM and (**b**) 5, 10, 20, and 30 RPM. In panel **a**, a 95% confidence interval is plotted with gray dashed lines. The confidence intervals are omitted in panel **b**. Within both plots, the “X” markers along the staircase show when a subject becomes censored (i.e., when a subject leaves the investigation and additional data are unavailable).

For each spin rate from 1 RPM to 32 RPM (i.e., the spin rate that applies 2 g loading at the feet and approximately 1 g at the rider’s center of mass for the shortest feasible centrifuge), we calculated the probability of individuals reaching each spin rate within each of one to 50 sessions. The entire compilation is provided as a table in the Supplementary Materials, while the plots in Fig. 3 provide the survival analysis staircase for (**a**) 20 RPM and (**b**) other example staircases at 5, 10, and 30 RPM. For each spin rate, as the number of days increases, there is a subsequent increase in the probability of subjects reaching the desired spin rate. For example, by training day 11 there is a 100% expected probability of subjects reaching 5 RPM, while this level of confidence requires several more training days for higher RPMs—about 29 days for 10 RPM and 41 days for 20 RPM. After the 50th day, there is a 70% probability that subjects would acclimate to 25 RPM and a 60% probability of reaching 30 RPM, due to the greater CC illusion stimulus associated with higher spin rates.

## Discussion

The results of this study show that all subjects continued to acclimate to the CC illusion with continued incremental exposure. Based on our predetermined criteria, the data show no evidence of subjects reaching a plateau in ending threshold within the duration investigated, and all subjects exhibited a non-zero (positive) acclimation rate, indicating an upward trend in tolerable threshold over multiple testing sessions. This suggests that there exists potential for any individual (even one who acclimates at a slower rate) to tolerably acclimate to a given spin rate of operational importance.

Acclimation occurred for all subjects while motion sickness was kept to a minimum. Throughout the study, subjects reported generally low motion sickness levels, and none of the subjects left the study due to nausea or dizziness, even those highly susceptible to the motion sickness that could be induced by the CC illusion. Notably, an individual’s average motion sickness level was correlated with the subject’s pretest MSSQ score. While this association might be expected, this is our first acclimation experiment in which the correlation was statistically significant. We suggest this finding resulted from the extended CC illusion testing and exposure, which allowed for a more representative average motion sickness rating for each subject.

Acclimation findings and motion sickness reports were comparable across our previous 10-day personalized study^14^ and the current extended study (Fig. 1a). This suggests that there was not a dramatic effect of our modifications to the staircase (including the catch trials) or a change in provocativeness over the additional sessions. Additionally, our largely male subject group in the current study and balanced cohort in the previous investigation performed comparably, further suggesting the absence of a gender effect in acclimation training. Because there were no significant differences between the results of these subject groups, we pooled subject data from both studies to complete the survival analysis.

By conducting survival analysis of the data from all 21 subjects exposed to a personalized, incremental training protocol, we aimed to create a tool to aid in development of centrifuge designs. This tool may provide designers with a human tolerability trade (i.e., allowable spin rate) to accompany existing engineering trades (e.g., mass, power, and volume). These results specifically reflect acclimation rates and performance using the protocol implemented in the current investigation; different acclimation approaches may result in different survival analysis estimates. Further, using the outcomes in Fig. 3 and the Supplementary Materials table, we note that the survival analysis estimates are likely not the upper limit for tolerability. In our protocol, we defined subjects to have not fully “acclimated” to a spin rate until they did not feel any CC illusion. Presumably, some presence of the illusion would still be tolerable for astronauts. This means that there may be a higher probability of subjects tolerating a given spin rate with fewer days of training, or for higher spin rates to become tolerable with a shorter training protocol. Therefore, designers should note that the analysis presented here likely provides a conservative estimate of tolerability.

Throughout the study, it was critically important that subjects remained naïve to the protocol and were able to maintain consistent criteria for reporting the presence of the CC illusion. We used blinded catch trials (presented at 50% or at least 5 RPM higher or lower than the subject’s current, near-threshold spin rate) to assess the reliability of subject reports and guard against the potential for subjects to report what they thought they should have been feeling or what they thought the test operators were expecting. Nearly 95.5% of all catch trials resulted in subjects reporting the presence or absence of the illusion as expected. The remaining 4.5% may be explained by factors outside of our control. Subjects verbally reported that at times, they could sense acceleration of the centrifuge, potentially from airflow or vibration cues (white noise was played to mask auditory cues and the acceleration rate was intended to be subthreshold). When subjects reported feeling a change in spin rate, they were often not sure if they were spinning up or spinning down. If, for example, a subject was exposed to a Low Catch Trial, but the vibration of the HERD made them mistakenly feel as though they were spinning up, they may report an unexpected presence of the illusion due to what they thought they should feel. Due to the infrequency of these unexpected results, we believe subjects were largely reliable in their reporting.

A more thorough discussion on the limitations of the experimental design and setup can be found elsewhere^14^, but briefly, it is important to note the most relevant limitations here. First, subjects were seated upright while spinning about an Earth-vertical axis; head tilts were performed in one axis (roll tilt) while spinning in one direction. As this was the first study completed of this extended duration, the setup allowed for greater confidence in our design due to reduced confounds. By targeting one head tilt axis and rotation direction, we were able to isolate the stimulus-response relationship and quantify acclimation in that single axis. Although the CC illusion acclimation appears to not readily transfer to other rotation axes^17^, we suggest these findings provide a proof-of-concept demonstration for short-radius centrifuge design, redefining previously accepted tolerable spin rate limits.

Second, we collected only subjects’ verbal reports of the presence or absence of the CC illusion following each head tilt. In contrast with recording vestibulo-ocular reflex eye movements, we recognize that this is a subjective measure. However, we focused on subjective perception, as the associated disorientation is likely to be the primary operational concern of the CC illusion for short-radius centrifugation. Despite the use of this subjective metric, our catch trial results suggest that subjects did report reliably.

As an additional limitation, all testing was completed in a ground-based, 1 g environment. It is unknown how CC illusion acclimation would be altered in microgravity on-orbit. Previous parabolic flight experiments producing ~20 s of microgravity^18–20^ and on-orbit experiments during Skylab^21–23^ suggest that in microgravity, the CC illusion may be less intense, but still present in some form. However, it is worth noting that these flight experiments had the subject’s head aligned with the spin axis (upright yaw rotating chair). Any centrifuge orientation in which the subject’s head is situated off-axis would induce centrifugal loading to the vestibular system. We speculate that this would create Earth-like sensory conflict as a result of the CC illusion. Additionally, it may be important to have a centrifuge on the surface of the Moon or Mars to continue to mitigate physiological deconditioning during long-duration surface stays. Any planetary body-based centrifuge would still have a gravitational force acting on the rider. Even if this was a fraction of Earth’s gravity (e.g., 1/6 g on the Moon or 3/8 g on Mars), it may still be sufficient to induce disorientation and motion sickness from the CC illusion like in our ground-based studies. The overall relevance of the CC illusion in both ground- and microgravity-based centrifuges drives the need for individuals to become more tolerant of the illusion in order to increase both the feasibility and tolerability of using AG as a spaceflight countermeasure.

Although we recognize the limitations of this ground-based approach for spaceflight, we also note that the encouraging results of our approach using an incremental, personalized protocol to induce acclimation to the CC illusion may have more broad applications to other vestibular adaptation and rehabilitation programs here on Earth. For example, previous research has shown that dynamic incremental training is a beneficial rehabilitation tool in improving gaze stabilizing reflexes^24^ and reducing postural instability^25^.

As a final limitation of the study, we note the challenge in recruiting subjects to participate in a study of such length. Only eight of our 11 enrolled subjects completed the investigation by meeting one of our ending criteria. Subjects were provided monetary incentives to finish the study, but in some cases, this was insufficient to overcome scheduling challenges. We note that the three subjects who left the study before reaching our ending criteria (X’s in Fig. 1b) appeared to be acclimating on similar trajectories as those subjects who did reach the ending criteria.

We recommend that future investigations work to test additional subjects and further validate the design tool created here, though in more operationally relevant configurations. Although we do not anticipate conflicting results, we believe it is necessary to ensure the efficacy of this protocol during off-axis centrifugation, in which the subjects lay supine on an off-axis rotating bed (i.e., conventional centrifuge configuration) rather than spinning about an Earth-vertical axis on an upright chair. Additionally, although we have demonstrated the prevalence of acclimation to head tilts performed in a rotating environment, subsequent studies should further investigate the potential for more the generalized acclimation that will be necessary for astronauts—acclimation to complex head movements or acclimation transfer across head tilt planes.

In this investigation, we sought to better understand human acclimation to the CC illusion through an extended personalized acclimation protocol, such that we could better inform conceptual centrifuge design. The results from this study quantify the longest CC illusion acclimation investigation completed to date. Although limited in sample size, our findings suggest that all individuals may have the capacity to acclimate to a spin rate of operational interest, if they are given a training protocol of sufficient duration. This leads us to believe that the CC illusion and/or individual motion sickness susceptibility may no longer be the limitations restricting certain short-radius AG approaches. Instead, future limitations of minimum radius designs may shift to subject height (the centrifuge will presumably not be shorter than the subject), engineering or power constraints, and/or other physiological concerns. We have shown that tolerable short-radius, fast-rotation centrifugation is possible, and have developed a design tool to quantify the expected acclimation to specific spin rates over up to 50 training sessions. This tool aims to assist in the development of a short-radius, intermittent centrifuge for artificial gravity implementation to enable superior protection of astronauts during long-duration space exploration.

## Methods

### Subjects

Eleven healthy subjects (10 M/1 F) volunteered to participate in this investigation. We did not intentionally recruit a cohort with more males; however, our previous studies with a balanced cohort^14^ suggest there are not significant gender effects. Subjects had an average age of 22.2 years old (range: 20–25 years) and, on average, scored in the 43rd percentile on the Motion Sickness Susceptibility Questionnaire^26^ (range: 0–89). Subjects were neither excluded nor included based on susceptibility to motion sickness; however, we verbally ensured that all subjects who were enrolled in the study had no known history of vestibular dysfunction. All subjects signed a written informed consent, and all protocols were approved by the University of Colorado Institutional Review Board.

### Equipment

This experiment utilized the upright chair configuration of the Human Eccentric Rotator Device (HERD) within the Bioastronautics Laboratory at the University of Colorado Boulder. As in previous studies^14–16^, subjects were positioned in the center of the rotating platform, seated in the chair with a 4-point harness, and spun clockwise in yaw about an Earth-vertical axis. All testing was completed in the dark. Head tilts were limited by two foam blocks on either side of the subject’s head, and earbuds played white noise to mask auditory cues from the HERD. Wireless two-way communication and video surveillance ensured continuous monitoring. Subjects verbally reported the presence of the CC illusion and their motion sickness ratings when prompted; they also pressed wireless pushbuttons for redundant reporting.

### Procedure

All subjects in this investigation were exposed to a threshold-based, personalized, incremental acclimation protocol. To accomplish this, we incremented spin rate based upon subject response to the CC illusion stimulus. Each subject experienced unique stimuli throughout the study, as each protocol was individualized to ensure maximal tolerability by exposing subjects to spin rates at or just above their threshold (i.e., barely perceivable). Critically, subjects remained naïve to the protocol and staircase as described. Subjects were informed that the investigation was a “CC illusion acclimation study”, and that the HERD may accelerate or decelerate at random times throughout the experiment, but they were to simply tilt their head when prompted, then report presence or absence of the CC illusion as a result of the head tilt without focusing much thought on the spin rate at which they were rotating. They were not made aware when the spin rate was changed nor the rules for why they may change.

All subjects began the investigation spinning at a supra-threshold spin rate of 10 RPM such that they could become familiar with the sensation of the CC illusion. Our previous investigations suggest that 10 RPM is a sufficiently strong stimulus to elicit the illusion in all subjects^14,15^. After spinning at 10 RPM for ~30 s (to allow for the equilibration of the endolymph in subjects’ inner ears), subjects performed a head tilt 40° (right ear) down over approximately one second and remained in the tilted position while reporting the presence or absence of the CC illusion as a direct result of that head tilt. Subjects verbally reported “yes I felt the illusion” or “no, I did not feel anything different from tilting my head in a stationary environment” while also pressing the corresponding pushbutton for reporting redundancy. After spinning with their head tilted for ~30 s, subjects were instructed to tilt their head back to the upright position over approximately one second and again report the presence or absence of the illusion. Following each head tilt pair (head tilt down and back to upright), the experiment operators decided whether to increase, maintain, or decrease the current spin rate, based on both subject response and our predetermined bidirectional staircase rules.

#### Protocol staircase

We employed an acclimation staircase that used each subject’s reporting of the CC illusion to determine the spin rate progression as the subject acclimated. The staircase was closely modeled after that which we used previously^14^, inspired by Cheung et al^13^. It sought to provide subjects with a CC illusion stimulus at or just above their threshold. The intention was to provide sufficient conflict between expected sensations and actual sensory input to drive acclimation, while limiting motion sickness (as compared to previous approaches cited earlier) by not being excessively provocative.

As in our previous staircase^14^, subjects were introduced to the stimulus at a supra-threshold spin rate of 10 RPM, administered only on the first day of the experiment. Subjects were accelerated to 10 RPM over 45 s, completed one head tilt pair at 10 RPM, then were decelerated to 1 RPM over 60 s to begin the training protocol. At each subsequent spin rate, if subjects reported not feeling the illusion on both head tilts of one head tilt pair, the spin rate was increased by 1 RPM over 10 s (acceleration was chosen in an effort to maintain naivety of the staircase and protocol in subjects). Alternatively, if subjects reported that they felt the illusion on either or both head tilts within one head tilt pair, the spin rate was nominally maintained. However, as an addition to our previous staircase^14^, in this study if subjects reported feeling the illusion on each head tilt of three consecutive head tilt pairs, the spin rate was decreased by 1 RPM over 10 s. If subjects repeatedly reported feeling the CC illusion (as would be true in this scenario), it would indicate that the stimulus was decidedly supra-threshold. We added this third option to enable the staircase to be bidirectional (i.e., the spin rate could decrease as well as increase). This addition is important to quantify extended acclimation and identify a plateau if it exists.

For the entire duration of the training protocol, subjects were accelerated or decelerated based upon their response to the previous stimuli, then spun for 30 s at the constant rate before any head tilts were performed. As done previously^14^, each subsequent session’s initial spin rate was 1 RPM less than the spin rate in which the subject first reported feeling the illusion on the prior session. Substantial effort was put forth to train individual subjects at roughly the same time each day (i.e., within the same 2–3 h), although subject availability ultimately dictated scheduling.

#### Catch trials

As subjects’ responses defined the staircase, a lack of proper reporting (in terms of subjects not responding truthfully or reliably regarding their perception of a presence or absence of the illusion) could result in inaccurate or incomplete results. To assess the reliability of subject responses, we added catch trials into the protocol, to which the subjects were naïve. On a catch trial (one trial is synonymous with one head tilt pair) we altered the spin rate outside of the standard staircase described above. Each catch trial was randomly selected as either a High Catch Trial (spin rate was adjusted to 50% higher than the spin rate at which the subject was exposed immediately prior to the catch trial) or a Low Catch Trial (50% lower than preceding spin rate). On High Catch Trials, we would expect subjects to report feeling the illusion on one or both head tilts of the head tilt pair, since the spin rate stimulus is much greater. Conversely, on a Low Catch Trial, we would expect that they would report not experiencing the illusion on one or both head tilts of the pair. Depending upon the previous spin rate, it was not always possible to increase or decrease the spin rate by exactly 50%, so we applied the following rules for catch trials and spin rate determination. First, the HERD can only be commanded with whole-number RPMs; therefore, if the desired 50% higher or 50% lower rate resulted in a decimal, Low Catch Trial spin rates were rounded down to the nearest whole number, while High Catch Trial spin rates were rounded up. Second, to ensure a sufficient adjustment to the illusion intensity, we required the change in spin rate between the regular protocol and the catch trial to be at least 5 RPM. The resulting catch trial spin rate could neither be slower than 1 RPM nor faster than 30 RPM (due to our safety protocol). If these rules were violated, the catch trial was not performed for that session. One catch trial was administered at a random time point within each session (one catch trial of the ~20 trials within each training session) to investigate if subjects were reporting as expected. Using catch trials to assess reporting reliability is particularly important given the duration of this study. Because subjects are testing for up to 50 sessions, subjects must maintain stable decision criteria from their initial distinct, supra-threshold CC illusion exposure at the beginning of the first session to the end of their participation in the investigation.

#### Ending criteria

Subjects completed one 25-min acclimation session on every weekday for at least 10 days and up to 50 total days. Unlike our initial personalized acclimation protocol^14^, all subjects did not complete the study after the same number of sessions. Instead, each subject remained in the experiment until one of three ending criteria were met:

1. Reaching a beginning threshold of 25 RPM
2. Reaching a plateau in acclimation (not increasing ending threshold for 10 consecutive sessions)
3. Completing 50 acclimation sessions

The ending criteria, to which the subjects remained naïve, were intended to maximize knowledge gained from the experiment while minimizing unnecessary subject involvement and/or subject risk. We hypothesized that if all subjects completed all 50 sessions, some subjects would likely reach spin rates that would exceed those of operational relevance. Although the optimal loading level is still an unknown design parameter, existing AG conceptual designs recommend loading on the order of 1 g at the subject’s center of mass and 2 g at the subject’s feet. Similarly, an optimized centrifuge size has not yet been determined; however, the centrifuge radius would likely be at least 2 m in order to accommodate even the tallest astronauts. Given these bounds, the fastest an operational centrifuge would be spun is around 30–35 RPM; acclimating to higher spin rates is likely unnecessary.

This rationale drove the development of our ending criteria. We stopped subjects after they achieved a sufficiently fast beginning threshold, defined as the fastest spin rate at which no illusion was felt at the beginning of the session (i.e., without any training during that session). A beginning threshold ending criteria of 25 RPM was selected to correspond to the spin rate required to create at least 1 g loading at the feet of the majority of our subjects (if they were to be positioned supine on a centrifuge with their head at the center of the centrifuge). Once subjects reached that beginning threshold cutoff, we tested them throughout the rest of that session; upon conclusion of the session, their participation in the study was terminated.

The second ending criteria was included in the event that continued acclimation was not possible with continued exposure—that subjects’ ending thresholds (i.e., fastest spin rate at which no illusion was felt at the end of a session) reached a plateau. If this was the case, we did not want to continue testing individuals when additional acclimation would not be possible.

Finally, if subjects did not reach a beginning threshold of 25 RPM or a plateau in ending threshold, we tested them for 50 total acclimation sessions. These ending criteria and testing duration allowed us to test our hypotheses regarding extended acclimation, working towards a better understanding of the capacity of subjects to acclimate to the CC illusion, thus informing centrifuge conceptual design.

#### Motion sickness monitoring

To verify that our protocol facilitated benign acclimation to the CC illusion, we asked subjects to verbally report their subjective motion sickness rating (MSR) once every 5 min of each 25-min acclimation session. Motion sickness scores were to be reported on the simple but commonly used scale of 0–20^6–9,11–13,17^, where a score of 0 is used to convey no sense of motion sickness, and a score of 20 represents the subject feeling as though he/she is on the verge of vomiting.

If at any point subjects reported that they were feeling ill or wanted to spin down (for motion sickness reasons or otherwise), the testing operators did so. Additionally, if a subject reported an MSR of 10/20 or higher, the day’s session would prematurely conclude. If the same subject reported 10/20 or higher on a subsequent session, he/she would not continue the study. This motion sickness-based ending criterion was implemented to prevent excessive motion sickness, though as discussed in the Results, no subject reported motion sickness greater than 10/20 more than once.

### Data and analysis

#### Metrics and variables of interest

We extracted similar metrics as in our initial 10-day acclimation study^14^, as both investigations sought to evaluate the tolerability and feasibility of acclimation. To assess feasibility of acclimation, we calculated beginning and ending threshold for each session. From these, we calculated a linear acclimation rate (a measure of the amount of acclimation achieved per session of testing). To quantify tolerability, we calculated the maximum and average motion sickness rating throughout all sessions and subjects. Each individual’s Motion Sickness Susceptibility Questionnaire (MSSQ) percentile was correlated with reported motion sickness levels.

With these variables, we were primarily interested in determining the long-term potential of subjects to acclimate to the CC illusion, and if the protocol changes implemented in the extended acclimation study (i.e., updated staircase and addition of catch trials) had a significant impact on how subjects were acclimating.

#### Statistical tests

We performed statistical tests in MATLAB and R/RStudio. The assumption of normality was verified with Anderson–Darling and Shapiro–Wilks tests, and F-tests were used to verify equality of variance. Comparisons between groups were performed using two-tailed, two-sample or paired *t*-tests with either equal or unequal variances (based on the result from the associated F-test). In the event that the dataset failed the normality tests, nonparametric tests were utilized (Mann–Whitney–Wilcoxon test). Finally, Spearman rank nonparametric correlation tests were used to measure potential association between subjects’ reported motion sickness levels and their pretest MSSQ percentiles. A required level of significance of $\alpha = 0.05$ was used for all statistical tests.

#### Use of survival analysis to develop design tool

To investigate the long-term outlook of acclimation—namely, the expected ability of subjects to acclimate to certain operationally relevant spin rates over a given number of testing days—we applied survival analysis to the collected data. To specifically accommodate subjects who did not complete the full investigation or did not reach a spin rate of operational interest, this statistical method was utilized to project the censored data forward in time^27^. Survival analysis works to critically evaluate the time it takes for an event to occur. The event of interest in the current investigation is reaching a given spin rate threshold of operational relevance, such as that required to reach a desired loading level with a given centrifuge size (e.g., 15 RPM for an 8-m diameter centrifuge). The ultimate goal with this analysis is to estimate a population acclimation curve from our sample.

We calculated the Kaplan–Meier survival analysis estimate^28^, which quantifies an approximation of “survival”, or the time until the event of interest occurs.

1 $$\hat S_t = \mathop {\prod }\limits_{t_i \le t} \left[ {1 - \frac{{d_i}}{{n_i}}} \right]$$

The survival rate is expressed with the survival function, *S*_*t*_, which is the proportion of individuals surviving longer than time *t* out of the total number of individuals studied at that time. The product limit method, unique to the Kaplan–Meier analysis, can be seen in Eq. 1. For our application, the survival function uses collected data to estimate the probability of not reaching the desired spin rate of interest over *t* number of sessions, where *t*_*i*_ refers to a time at which at least one subject reached the desired spin rate, *d*_*t*_ references the total number of subjects reaching the desired rate at that time, and *n*_*t*_ is the number of subjects who are still being tested and have not yet acclimated to the given spin rate. The complement of that percentage provides a calculation of the probability that subjects would reach the desired spin rate over *t* days of training, assuming the adoption of our protocol exposing subjects to one, 25-min session per day. For each desired spin rate, a staircase cumulative survival function (i.e., estimated population acclimation curve) can be plotted, showing how the probability of reaching the desired ending threshold increases as the number of training sessions increases. These developed curves can be used to aid in the conceptual design and development of a spaceflight centrifuge by ensuring tolerability of the selected design.

### Reporting Summary

Further information on research design is available in the Nature Research Reporting Summary linked to this article.

## Supplementary information

Survival Analysis Complete Results

Reporting Summary Checklist FLAT

## Data Availability

The raw minimal datasets for this study have been made publicly available (https://osf.io/zw6xe/). We request citing this paper when using these datasets for further analysis.
