# Supplementary material for: Improved feasibility of astronaut short-radius artificial gravity through a 50-day incremental, personalized, vestibular acclimation protocol
Source: NPJ Microgravity. 2020 Aug 26;6:22. doi: 10.1038/s41526-020-00112-w (PMC7450067; doi:10.1038/s41526-020-00112-w)
Supplement: Supplementary file 1 — Survival Analysis Complete Results [file 41526_2020_112_MOESM1_ESM.pdf]

SUPPLEMENTARY INFORMATION

Survival Analysis Complete Results: This table provides the entire collection of results from our survival analysis of CC illusion acclimation training. In the table, each cell contains the probability (in %) of subjects acclimating to the spin rate of interest (shown along the top row) over the given number of training days (shown in the leftmost column). Accompanying this probability within each cell is the corresponding 95% confidence interval. As seen in the table, a longer duration of testing is required to reach a higher probability of individuals acclimating to faster spin rates. We intend for these tables to be used as a reference by centrifuge designers when evaluating tradeoffs of tolerable spin rate and other design variables (centripetal acceleration, radius, mass, power, and volume).

Training Day	1 RPM	2 RPM	3 RPM	4 RPM	5 RPM	6 RPM	7 RPM	8 RPM	9 RPM	10 RPM
1	100	81 [54-92]	67 [39-82]	62 [34-78]	43 [17-61]	33 [10-51]	19 [0-34]	10 [0-21]	0	0
2		95 [68-99]	86 [59-95]	76 [49-89]	67 [39-82]	52 [25-70]	43 [17-61]	38 [13-56]	24 [3-40]	14 [0-28]
3		100	86 [59-95]	76 [49-89]	67 [39-82]	62 [34-78]	52 [25-70]	43 [17-61]	38 [13-56]	33 [10-51]
4			95 [68-99]	81 [54-92]	76 [49-89]	67 [39-82]	62 [34-78]	57 [30-74]	52 [25-70]	38 [13-56]
5			95 [68-99]	86 [59-95]	76 [49-89]	76 [49-89]	71 [44-85]	62 [34-78]	57 [30-74]	48 [21-65]
6			95 [68-99]	90 [64-97]	81 [54-92]	76 [49-89]	71 [44-85]	67 [39-82]	67 [39-82]	52 [25-70]
7			95 [68-99]	95 [68-99]	86 [59-95]	81 [54-92]	76 [49-89]	76 [49-89]	76 [49-89]	71 [44-85]
8			100	95 [68-99]	90 [64-97]	81 [54-92]	76 [49-89]	76 [49-89]	76 [49-89]	76 [49-89]
9				95 [68-99]	90 [64-97]	81 [54-92]	81 [54-92]	76 [49-89]	76 [49-89]	76 [49-89]
10				95 [68-99]	90 [64-97]	81 [54-92]	81 [54-92]	76 [49-89]	76 [49-89]	76 [49-89]
11					90 [64-97]	94 [61-99]	81 [54-92]	76 [49-89]	76 [49-89]	76 [49-89]
12					100	94 [61-99]	81 [54-92]	76 [49-89]	76 [49-89]	76 [49-89]
13						100	87 [58-96]	76 [49-89]	76 [49-89]	76 [49-89]
14							87 [58-96]	76 [49-89]	76 [49-89]	76 [49-89]
15							94 [61-99]	84 [52-95]	76 [49-89]	76 [49-89]
16							94 [61-99]	84 [52-95]	84 [52-94]	76 [49-89]
17							100	84 [52-95]	84 [52-94]	76 [49-89]
18								84 [52-95]	84 [52-94]	76 [49-89]
19								92 [53-99]	84 [52-94]	76 [49-89]
20								100	84 [52-94]	76 [49-89]
21									84 [52-94]	84 [52-95]
22									84 [52-94]	84 [52-95]
23									92 [53-99]	84 [52-95]
24									92 [53-99]	84 [52-95]
25									92 [53-99]	92 [53-99]
26									92 [53-99]	92 [53-99]
27									92 [53-99]	92 [53-99]
28									100	100
29										
30										
31										
32										
33										
34										
35										
36										
37										
38										
39										
40										
41										
42										
43										
44										
45										
46										
47										
48										
49										
50										

11 RPM	12 RPM	13 RPM	14 RPM	15 RPM	16 RPM	17 RPM	18 RPM	19 RPM	20 RPM	21 RPM	22 RPM	23 RPM	24 RPM	25 RPM	26 RPM	27 RPM
0	0	0	0	0	0	0	0	0	0	0	0	0	0	0	0	0
5 [0-13]	0	0	0	0	0	0	0	0	0	0	0	0	0	0	0	0
19 [0-34]	19 [0-34]	14 [0-28]	14 [0-28]	5 [0-13]	0	0	0	0	0	0	0	0	0	0	0	0
33 [10-51]	33 [10-51]	24 [3-40]	19 [0-34]	10 [0-21]	10 [0-21]	10 [0-21]	5 [0-13]	5 [0-13]	5 [0-13]	5 [0-13]	0	0	0	0	0	0
43 [17-61]	43 [17-61]	38 [13-56]	19 [0-34]	19 [0-34]	14 [0-28]	10 [0-21]	10 [0-21]	10 [0-21]	10 [0-21]	10 [0-21]	10 [0-21]	5 [0-13]	5 [0-13]	5 [0-13]	0	0
43 [17-61]	43 [17-61]	43 [17-61]	38 [13-56]	29 [6-46]	29 [6-46]	24 [3-40]	14 [0-28]	10 [0-21]	10 [0-21]	10 [0-21]	10 [0-21]	10 [0-21]	10 [0-21]	10 [0-21]	5 [0-13]	5 [0-13]
57 [30-74]	52 [25-70]	48 [21-65]	43 [17-61]	33 [10-51]	33 [10-51]	33 [10-51]	29 [6-46]	19 [0-34]	14 [0-28]	14 [0-28]	10 [0-21]	10 [0-21]	10 [0-21]	10 [0-21]	10 [0-21]	10 [0-21]
62 [34-78]	62 [34-78]	62 [34-78]	57 [30-74]	57 [30-74]	33 [10-51]	33 [10-51]	33 [10-51]	29 [6-46]	24 [3-40]	19 [0-34]	14 [0-28]	10 [0-21]	10 [0-21]	14 [0-28]	10 [0-21]	10 [0-21]
71 [44-85]	67 [39-82]	67 [39-82]	67 [39-82]	57 [30-74]	48 [21-65]	38 [13-56]	33 [10-51]	33 [10-51]	29 [6-46]	24 [3-40]	24 [3-40]	24 [3-40]	19 [0-34]	19 [0-34]	10 [0-21]	10 [0-21]
76 [49-89]	71 [44-85]	67 [39-82]	67 [39-82]	67 [39-82]	52 [25-70]	48 [21-65]	38 [13-56]	38 [13-56]	33 [10-51]	33 [10-51]	29 [6-46]	24 [3-40]	24 [3-40]	24 [3-40]	19 [0-34]	14 [0-28]
76 [49-89]	71 [44-85]	73 [44-87]	73 [44-87]	67 [39-82]	52 [25-70]	48 [21-65]	38 [13-56]	38 [13-56]	33 [10-51]	33 [10-51]	29 [6-46]	24 [3-40]	24 [3-40]	24 [3-40]	19 [0-34]	14 [0-28]
76 [49-89]	71 [44-85]	73 [44-87]	73 [44-87]	67 [39-82]	52 [25-70]	48 [21-65]	47 [17-66]	47 [17-66]	50 [17-70]	42 [13-61]	44 [14-64]	32 [6-51]	32 [6-51]	32 [6-51]	28 [2-47]	24 [0-43]
76 [49-89]	71 [44-85]	73 [44-87]	73 [44-87]	73 [44-87]	52 [25-70]	48 [21-65]	56 [21-75]	56 [21-75]	58 [23-78]	42 [13-61]	44 [14-64]	41 [10-61]	32 [6-51]	32 [6-51]	28 [2-47]	24 [0-43]
76 [49-89]	79 [48-91]	80 [49-92]	80 [49-92]	80 [49-92]	71 [34-88]	56 [25-75]	65 [27-83]	65 [27-83]	58 [23-78]	58 [23-78]	60 [24-79]	49 [14-70]	41 [10-61]	32 [6-51]	28 [2-47]	24 [0-43]
76 [49-89]	79 [48-91]	80 [49-92]	80 [49-92]	80 [49-92]	71 [34-88]	56 [25-75]	65 [27-83]	65 [27-83]	58 [23-78]	58 [23-78]	60 [24-79]	49 [14-70]	51 [14-72]	41 [10-63]	38 [5-60]	24 [0-43]
76 [49-89]	79 [48-91]	80 [49-92]	80 [49-92]	80 [49-92]	71 [34-88]	56 [25-75]	65 [27-83]	65 [27-83]	58 [23-78]	58 [23-78]	60 [24-79]	49 [14-70]	51 [14-72]	41 [10-63]	38 [5-60]	24 [0-43]
76 [49-89]	79 [48-91]	80 [49-92]	80 [49-92]	80 [49-92]	71 [34-88]	65 [30-83]	65 [27-83]	65 [27-83]	58 [23-78]	58 [23-78]	60 [24-79]	49 [14-70]	51 [14-72]	51 [14-72]	49 [10-71]	35 [1-57]
76 [49-89]	79 [48-91]	80 [49-92]	80 [49-92]	80 [49-92]	71 [34-88]	65 [30-83]	65 [27-83]	65 [27-83]	67 [29-84]	58 [23-78]	60 [24-79]	49 [14-70]	51 [14-72]	51 [14-72]	49 [10-71]	46 [6-69]
76 [49-89]	79 [48-91]	80 [49-92]	80 [49-92]	80 [49-92]	71 [34-88]	74 [36-89]	73 [34-89]	65 [27-83]	67 [29-84]	67 [29-84]	60 [24-79]	49 [14-70]	51 [14-72]	51 [14-72]	49 [10-71]	46 [6-69]
76 [49-89]	79 [48-91]	80 [49-92]	80 [49-92]	80 [49-92]	71 [34-88]	74 [36-89]	73 [34-89]	73 [34-89]	67 [29-84]	67 [29-84]	60[24-79]	49 [14-70]	51 [14-72]	51 [14-72]	49 [10-71]	46 [6-69]
76 [49-89]	79 [48-91]	80 [49-92]	80 [49-92]	80 [49-92]	71 [34-88]	74 [36-89]	73 [34-89]	73 [34-89]	67 [29-84]	67 [29-84]	60[24-79]	49 [14-70]	51 [14-72]	51 [14-72]	49 [10-71]	46 [6-69]
76 [49-89]	79 [48-91]	80 [49-92]	80 [49-92]	80 [49-92]	71 [34-88]	74 [36-89]	73 [34-89]	73 [34-89]	75 [36-90]	75 [36-90]	76 [38-91]	59 [20-79]	60 [20-81]	51 [14-72]	49 [10-71]	46 [6-69]
84 [52-95]	79 [48-91]	80 [49-92]	80 [49-92]	80 [49-92]	71 [34-88]	74 [36-89]	73 [34-89]	73 [34-89]	75 [36-90]	75 [36-90]	76 [38-91]	59 [20-79]	60 [20-81]	51 [14-72]	49 [10-71]	46 [6-69]
84 [52-95]	79 [48-91]	80 [49-92]	80 [49-92]	80 [49-92]	71 [34-88]	74 [36-89]	73 [34-89]	73 [34-89]	75 [36-90]	75 [36-90]	76 [38-91]	59 [20-79]	60 [20-81]	60 [20-81]	59 [16-80]	56 [12-78]
84 [52-95]	79 [48-91]	80 [49-92]	80 [49-92]	80 [49-92]	71 [34-88]	74 [36-89]	73 [34-89]	73 [34-89]	75 [36-90]	75 [36-90]	76 [38-91]	59 [20-79]	60 [20-81]	60 [20-81]	59 [16-80]	56 [12-78]
84 [52-95]	86 [53-96]	80 [49-92]	80 [49-92]	80 [49-92]	71 [34-88]	74 [36-89]	73 [34-89]	73 [34-89]	75 [36-90]	75 [36-90]	76 [38-91]	70 [26-87]	70 [27-88]	70 [27-88]	59 [16-80]	56 [12-78]
92 [53-99]	93 [56-99]	80 [49-92]	80 [49-92]	80 [49-92]	71 [34-88]	74 [36-89]	73 [34-89]	73 [34-89]	75 [36-90]	75 [36-90]	76 [38-91]	70 [26-87]	70 [27-88]	70 [27-88]	59 [16-80]	56 [12-78]
92 [53-99]	93 [56-99]	90 [47-98]	80 [49-92]	80 [49-92]	71 [34-88]	74 [36-89]	73 [34-89]	73 [34-89]	75 [36-90]	75 [36-90]	76 [38-91]	70 [26-87]	70 [27-88]	70 [27-88]	59 [16-80]	56 [12-78]
92 [53-99]	93 [56-99]	90 [47-98]	90 [47-98]	80 [49-92]	71 [34-88]	74 [36-89]	73 [34-89]	73 [34-89]	75 [36-90]	75 [36-90]	76 [38-91]	70 [26-87]	70 [27-88]	70 [27-88]	73 [20-91]	71 [16-90]
92 [53-99]	93 [56-99]	90 [47-98]	90 [47-98]	80 [49-92]	71 [34-88]	74 [36-89]	73 [34-89]	73 [34-89]	75 [36-90]	75 [36-90]	76 [38-91]	70 [26-87]	70 [27-88]	70 [27-88]	73 [20-91]	71 [16-90]
92 [53-99]	93 [56-99]	90 [47-98]	90 [47-98]	80 [49-92]	71 [34-88]	74 [36-89]	73 [34-89]	73 [34-89]	75 [36-90]	75 [36-90]	76 [38-91]	70 [26-87]	70 [27-88]	70 [27-88]	73 [20-91]	71 [16-90]
100	93 [56-99]	90 [47-98]	90 [47-98]	90 [47-98]	86 [28-97]	87 [32-97]	73 [34-89]	73 [34-89]	75 [36-90]	75 [36-90]	76 [38-91]	70 [26-87]	70 [27-88]	70 [27-88]	73 [20-91]	71 [16-90]
	93 [56-99]	90 [47-98]	90 [47-98]	90 [47-98]	86 [28-97]	87 [32-97]	73 [34-89]	73 [34-89]	75 [36-90]	75 [36-90]	76 [38-91]	70 [26-87]	70 [27-88]	70 [27-88]	73 [20-91]	71 [16-90]
	100	90 [47-98]	90 [47-98]	90 [47-98]	86 [28-97]	87 [32-97]	73 [34-89]	73 [34-89]	75 [36-90]	75 [36-90]	76 [38-91]	70 [26-87]	70 [27-88]	70 [27-88]	73 [20-91]	71 [16-90]
							73 [34-89]	73 [34-89]	75 [36-90]	75 [36-90]	76 [38-91]	70 [26-87]	70 [27-88]	70 [27-88]	73 [20-91]	71 [16-90]
							73 [34-89]	73 [34-89]	75 [36-90]	75 [36-90]	76 [38-91]	70 [26-87]	70 [27-88]	70 [27-88]	73 [20-91]	71 [16-90]
							73 [34-89]	73 [34-89]	75 [36-90]	75 [36-90]	76 [38-91]	70 [26-87]	70 [27-88]	70 [27-88]	73 [20-91]	71 [16-90]
							100	100	75 [36-90]	75 [36-90]	76 [38-91]	70 [26-87]	70 [27-88]	70 [27-88]	73 [20-91]	71 [16-90]
									75 [36-90]	75 [36-90]	76 [38-91]	70 [26-87]	70 [27-88]	70 [27-88]	73 [20-91]	71 [16-90]
									75 [36-90]	75 [36-90]	76 [38-91]	70 [26-87]	70 [27-88]	70 [27-88]	73 [20-91]	71 [16-90]
									100	100	76 [38-91]	70 [26-87]	70 [27-88]	70 [27-88]	73 [20-91]	71 [16-90]
											76 [38-91]	70 [26-87]	70 [27-88]	70 [27-88]	73 [20-91]	71 [16-90]
											76 [38-91]	70 [26-87]	70 [27-88]	70 [27-88]	73 [20-91]	71 [16-90]
											76 [38-91]	70 [26-87]	70 [27-88]	70 [27-88]	73 [20-91]	71 [16-90]
											76 [38-91]	70 [26-87]	70 [27-88]	70 [27-88]	73 [20-91]	71 [16-90]
											76 [38-91]	70 [26-87]	70 [27-88]	70 [27-88]	73 [20-91]	71 [16-90]
											100	70 [26-87]	70 [27-88]	70 [27-88]	73 [20-91]	71 [16-90]
												100	100	70 [27-88]	73 [20-91]	71 [16-90]
														70 [27-88]	73 [20-91]	71 [16-90]
														70 [27-88]	73 [20-91]	71 [16-90]

28 RPM	29 RPM	30 RPM	31 RPM	32 RPM
0	0	0	0	0
0	0	0	0	0
0	0	0	0	0
0	0	0	0	0
0	0	0	0	0
0	0	0	0	0
10 [0-21]	10 [0-21]	5 [0-13]	5 [0-13]	5 [0-13]
10 [0-21]	10 [0-21]	5 [0-13]	5 [0-13]	5 [0-13]
10 [0-21]	10 [0-21]	5 [0-13]	5 [0-13]	5 [0-13]
14 [0-28]	14 [0-28]	19 [0-34]	5 [0-13]	5 [0-13]
14 [0-28]	14 [0-28]	19 [0-34]	5 [0-13]	5 [0-13]
24 [0-43]	24 [0-43]	19 [0-34]	5 [0-13]	5 [0-13]
24 [0-43]	24 [0-43]	19 [0-34]	5 [0-13]	5 [0-13]
24 [0-43]	24 [0-43]	19 [0-34]	5 [0-13]	5 [0-13]
24 [0-43]	24 [0-43]	19 [0-34]	5 [0-13]	5 [0-13]
24 [0-43]	24 [0-43]	29 [1-49]	17 [0-37]	5 [0-13]
35 [1-57]	35 [1-57]	29 [1-49]	17 [0-37]	5 [0-13]
46 [6-69]	46 [6-69]	29 [1-49]	17 [0-37]	5 [0-13]
46 [6-69]	46 [6-69]	29 [1-49]	17 [0-37]	5 [0-13]
46 [6-69]	46 [6-69]	29 [1-49]	17 [0-37]	5 [0-13]
46 [6-69]	46 [6-69]	39 [5-61]	29 [0-53]	18 [0-41]
46 [6-69]	46 [6-69]	39 [5-61]	29 [0-53]	18 [0-41]
46 [6-69]	46 [6-69]	39 [5-61]	29 [0-53]	18 [0-41]
46 [6-69]	46 [6-69]	49 [10-72]	40 [0-65]	18 [0-41]
56 [12-78]	46 [6-69]	49 [10-72]	40 [0-65]	32 [0-58]
56 [12-78]	46 [6-69]	49 [10-72]	40 [0-65]	32 [0-58]
56 [12-78]	46 [6-69]	49 [10-72]	40 [0-65]	32 [0-58]
56 [12-78]	46 [6-69]	49 [10-72]	40 [0-65]	32 [0-58]
56 [12-78]	46 [6-69]	49 [10-72]	40 [0-65]	32 [0-58]
56 [12-78]	46 [6-69]	49 [10-72]	40 [0-65]	32 [0-58]
71 [16-90]	59 [10-81]	60 [17-80]	55 [2-80]	49 [0-76]
71 [16-90]	59 [10-81]	60 [17-80]	55 [2-80]	49 [0-76]
71 [16-90]	59 [10-81]	60 [17-80]	55 [2-80]	49 [0-76]
71 [16-90]	59 [10-81]	60 [17-80]	55 [2-80]	49 [0-76]
71 [16-90]	59 [10-81]	60 [17-80]	55 [2-80]	49 [0-76]
71 [16-90]	59 [10-81]	60 [17-80]	55 [2-80]	49 [0-76]
71 [16-90]	59 [10-81]	60 [17-80]	55 [2-80]	49 [0-76]
71 [16-90]	59 [10-81]	60 [17-80]	55 [2-80]	49 [0-76]
71 [16-90]	59 [10-81]	60 [17-80]	55 [2-80]	49 [0-76]
71 [16-90]	59 [10-81]	60 [17-80]	55 [2-80]	49 [0-76]
71 [16-90]	59 [10-81]	60 [17-80]	55 [2-80]	49 [0-76]
71 [16-90]	59 [10-81]	60 [17-80]	55 [2-80]	49 [0-76]
71 [16-90]	59 [10-81]	60 [17-80]	55 [2-80]	49 [0-76]
71 [16-90]	59 [10-81]	60 [17-80]	55 [2-80]	49 [0-76]
71 [16-90]	59 [10-81]	60 [17-80]	55 [2-80]	49 [0-76]
71 [16-90]	59 [10-81]	60 [17-80]	55 [2-80]	49 [0-76]
71 [16-90]	59 [10-81]	60 [17-80]	55 [2-80]	49 [0-76]
71 [16-90]	59 [10-81]	60 [17-80]	55 [2-80]	49 [0-76]
71 [16-90]	59 [10-81]	60 [17-80]	55 [2-80]	49 [0-76]
71 [16-90]	59 [10-81]	60 [17-80]	55 [2-80]	49 [0-76]
